# Towards prevention of acute lung injury: frequency and outcomes of emergency department patients at-risk – a multicenter cohort study

**DOI:** 10.1186/1865-1380-5-22

**Published:** 2012-05-27

**Authors:** Peter C Hou, Marie-Carmelle Elie-Turenne, Aya Mitani, Jonathan M Barry, Erica Y Kao, Jason E Cohen, Gyorgy Frendl, Ognjen Gajic, Nina T Gentile

**Affiliations:** 1 Department of Emergency Medicine, Brigham and Women’s Hospital, Boston, MA, USA; 2 Division of Burn, Trauma, and Surgical Critical Care, Brigham and Women’s Hospital, Boston, MA, USA; 3 Surgical Intensive Care Unit Translational Research (STAR) Center, Brigham and Women’s Hospital, Boston, MA, USA; 4 Harvard Medical School, Boston, MA, USA; 5 Department of Emergency Medicine, University of Florida College of Medicine, 1329 SW 16th Street, Gainesville FL 32610, USA; 6 Emergency Department, Shands University of Florida, Medical Center, Gainesville, FL, USA; 7 Department of Anesthesiology, Perioperative and Pain Medicine, Brigham and Women’s Hospital, Boston, MA, USA; 8 Department of Emergency Medicine, Albany Medical Center, Albany, NY, USA; 9 Albany Medical College, Albany, NY, USA; 10 Division of Pulmonary and Critical Care Medicine, Department of Medicine, Mayo Clinic, Rochester, MN, USA; 11 Multidisciplinary Epidemiology and Translational Research in Intensive Care (METRIC), Mayo Clinic, Rochester, MN, USA; 12 Mayo Medical School, Rochester, MA, USA; 13 Department of Emergency Medicine, Temple University Hospital, Philadelphia, PA, USA; 14 Temple University School of Medicine, Philadelphia, PA, USA; 15 Harvard Medical School, Department of Emergency Medicine & Division of Burn, Trauma, and Surgical Critical Care, Department of Surgery, Brigham and Women’s Hospital, 75 Francis Street, Neville House 312-B, Boston, MA 02115, USA; 16 Department of Medicine, Stanford Hospitals and Clinics, 300 Pasteur Drive, Room: S102, MC: 5110, Stanford, CA 94305, USA; 17 Division of Burn, Trauma, and Surgical Critical Care, Brigham and Women’s Hospital, 75 Francis Street, Boston, \ 02115, USA; 18 Department of Emergency Medicine, Brigham and Women’s Hospital, 75 Francis Street, Boston, MA 02115, USA; 19 Albany Medical Center Emergency Medicine Group, 47 New Scotland Avenue, MC 139, Albany, NY 12208, USA; 20 Department of Anesthesiology Perioperative and Pain Medicine, Brigham and Women's Hospital, 75 Francis Street, Boston, MA 02115, USA; 21 Pulmonary and Critical Care Medicine, Mayo Clinic, Old Marian Hall, Second Floor, Room 115, 200 First St. SW, Rochester, MN 5590, USA; 22 Department of Emergency Medicine, Temple University Hospital, Administrative Office, 10th Floor, Jones Hall, 1316 W. Ontario Street, Philadelphia, PA 19140, USA

## Abstract

****Background**:**

Few emergency department (ED) evaluations on acute lung injury (ALI) have been carried out; hence, we sought to describe a cohort of hospitalized ED patients at risk for ALI development.

****Methods**:**

Patients presenting to the ED with at least one predisposing condition to ALI were included in this study, a subgroup analysis of a multicenter observational cohort study (USCIITG-LIPS 1). Patients who met ALI criteria within 6 h of initial ED assessment, received end-of-life care, or were readmitted during the study period were excluded. Primary outcome was frequency of ALI development; secondary outcomes were ICU and hospital mortality.

****Results**:**

Twenty-two hospitals enrolled 4,361 patients who were followed from the ED to hospital discharge. ALI developed in 303 (7.0 %) patients at a median onset of 2 days (IQR 2–5). Of the predisposing conditions, frequency of ALI development was highest in patients who had aortic surgery (43 %) and lowest in patients with pancreatitis (2.8 %). Compared to patients who did not develop ALI, those who did had higher ICU (24 % vs. 3.0 %, *p* < 0.001) and hospital (28 % vs. 4.6 %, *p* < 0.001) mortality, and longer hospital length of stay (16 vs. 5 days, *p* < 0.001). Among the 22 study sites, frequency of ALI development varied from less than 1 % to more than 12 % after adjustment for APACHE II.

****Conclusions**:**

Seven percent of hospitalized ED patients with at least one predisposing condition developed ALI. The frequency of ALI development varied significantly according to predisposing conditions and across institutions. Further research is warranted to determine the factors contributing to ALI development.

##  Background

**Figure 1:**
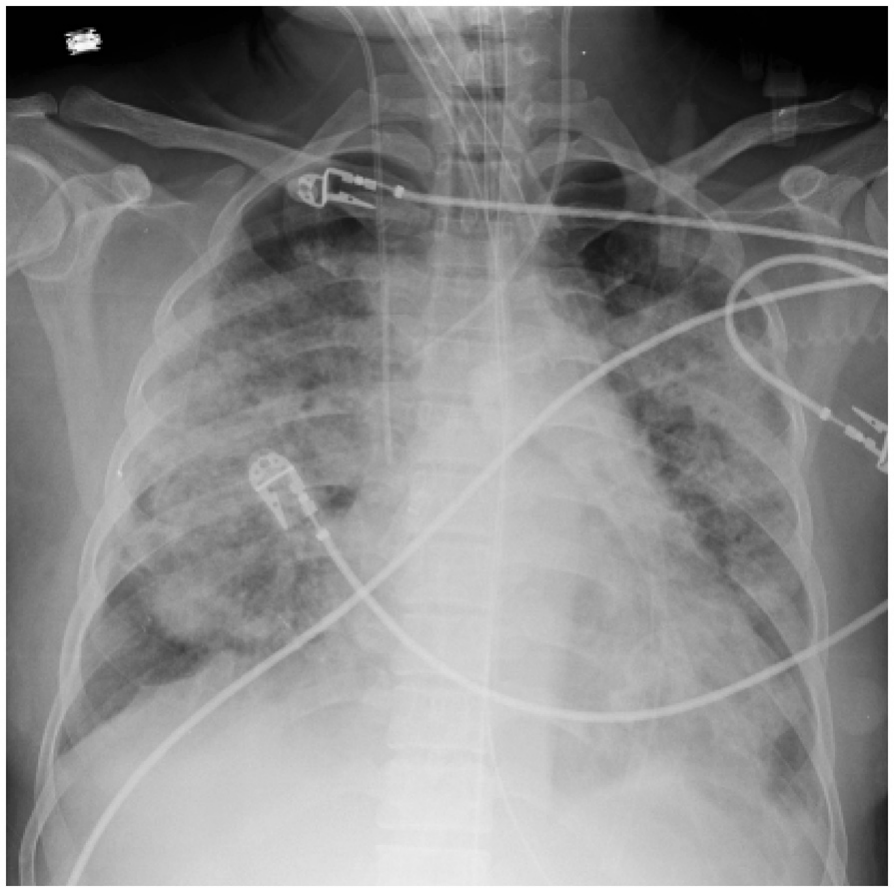
Chest x-ray representing acute lung injury.

Adult respiratory distress syndrome (ARDS) was first described by Ashbaugh [1] in 1967. Since then, the definition and criteria for this syndrome have evolved. At the 1994 American–European Consensus Conference (AECC), experts agreed to the following terminology: Acute lung injury (ALI) was defined as the acute onset of hypoxemia [PaO_2_/FiO_2_ (partial pressure of arterial oxygen/fractional concentration of inspired oxygen) ≤ 300 mmHg] and bilateral infiltrates on frontal chest x-ray (Figure 1), in the clinical absence of left atrial hypertension (or when measured, pulmonary-artery wedge pressure < 18 mm Hg) [2]. ARDS is the more severe form of ALI with hypoxemia at 200 mmHg or less [2-4]. In the US, approximately 150,000 to 190,600 cases of ALI occur annually, with an associated mortality rate of 38 to 44 %, 3.6 million hospital days, and long-term functional disabilities and cost after intensive care unit (ICU) discharge [5-8]. Pathophysiologically, ALI is classically characterized by an increased permeability of the alveolar-capillary membrane resulting in the influx of protein-rich edema fluid into the air spaces, and etiologies of ALI are myriad, including direct (pulmonary) and indirect (extrapulmonary) causes [3,4,9].

### **Importance**

Despite numerous randomized controlled trials, a lung protective strategy during mechanical ventilation remains the only therapy shown to improve survival in patients with established ALI [3,4,10-12]. According to the “two-hit” model of ALI development before ALI becomes clinically apparent, a pre-ALI state exists following the first injury to the lungs. The United States Critical Illness and Injury Trials Group-Lung Injury Prevention Study (USCIITG-LIPS 1) investigators focused on defining patient characteristics that would allow us to identify these patients before overt ALI develops. Interventions delivered in this early phase of care can offer potential prevention of ALI. As most clinical studies in ALI have primarily focused on mechanically ventilated patients, insight into a potentially preventable phase of ALI prior to its development is currently lacking [13,14]. Data suggest that ALI is rarely present at the time of initial Emergency Department (ED) evaluation; however, a search in top emergency medicine journals yielded many case reports of patients presenting with ALI [15-18]. In reality, many ED patients may have unrecognized ALI and possess many predisposing conditions for ALI development. Recently, a National Heart Lung Blood Institute Workshop Report on future clinical research in ALI recommended to continue the development of strategies to perform ALI prevention trials and observational studies of patients without ALI undergoing prolonged mechanical ventilation [19]. Following the paradigm of trauma team care for major trauma, activation of the cardiac catheterization laboratory team for ST-elevation myocardial infarction and acute stroke teams for ischemic stroke, and early goal-directed therapy for severe sepsis, clinical benefit may be derived from early identification of and preventative interventions for patients at risk of developing ALI.

### **Goal of this investigation**

We evaluated the frequency of ALI development in at-risk hospitalized ED patients among the study sites, described the predisposing conditions and risk modifiers of ALI development, and determined the attribution of ALI to hospital mortality.

## Methods

### **Study design**

This is a subgroup analysis of data from a multicenter, observational cohort study, the United States Critical Injury and Illness Trials Group-Lung Injury Prevention Study 1 (USCIITG-LIPS 1) [20,21]. All participating study sites received approval from their respective local institutional review board. The study flow diagram is illustrated in Additional file 1: Appendix 1.

### **Study setting**

From March through August 2009, 22 centers (20 American and 2 Turkish hospitals) enrolled patients with at least one ALI predisposition admitted from the ED. Patients were enrolled prospectively at 19 study sites and retrospectively at 3 sites.

### **Selection of participants**

Consecutive adult ED patients admitted to academic and community acute care hospitals were eligible for the study if they presented with one or more *a priori* defined conditions predisposing to ALI (shock, aspiration, sepsis, pancreatitis, pneumonia, high-risk trauma: traumatic brain injury, smoke inhalation, near drowning, lung contusion, multiple fractures; high-risk surgery: thoracic, spine, acute abdomen, cardiac, aortic vascular; and emergency surgery). Patients were excluded when ALI was present at initial assessment, if they were transferred from an in-patient setting, died in the ED, admitted for comfort or hospice care, or re-admitted during the study period. Hospital admission logs were reviewed to minimize the possibility that patients with predisposing conditions were missed. After identification of at-risk ED patients, they were followed through their hospitalization prospectively in 19 hospitals. In the three hospitals that enrolled retrospectively, investigators followed the same protocol and definitions, but data were collected after patient discharge.

### **Data collection and processing**

Baseline characteristics, including demographics, comorbidities, and clinical variables, were collected during the first 6 h of initial ED evaluation. Predisposing conditions and ALI risk modifiers were identified and collected. Predisposing conditions were pre-defined, and ALI risk modifiers included: alcohol abuse, obesity chemotherapy, diabetes mellitus, smoking, tachypnea, hypoxemia, oxygen supplementation, hypoalbuminemia, and acidosis.

De-identified subject information was entered at each center into the secure, password-protected NIH-supported web form (REDCap http://www.project-redcap.org). Electronic range checks and validation rules were utilized to eliminate erroneous data entry and artifacts in numeric values. Prior to study initiation at each site, investigators and study coordinators reviewed the definitions of each risk factor (see Additional file 1: Appendix 2) and received mandatory structured online training for ALI assessment. Briefly, in determining if a chest radiograph is consistent with ALI, assessment begins with interpretability of the x-ray followed by evaluation for bilateral opacities generally described as infiltrates consistent with pulmonary edema. Only when the bilateral opacities are not fully explained by non-qualifying opacities (i.e., pulmonary fibrosis) and not limited to the lower lung zones with normal parenchyma above is the chest radiograph consistent with ALI. In addition, a formal training session was provided during the 2009 USCIITG meeting in Nashville, TN. The principal investigators from each site were responsible for data collection and entry, as well as quality control.

### **Outcome measures**

The primary outcome was the development of AECC-defined ALI during the hospital admission of at-risk ED patients. Secondary outcomes included time to ALI development; proportion and duration of invasive and non-invasive mechanical ventilation; vasopressor requirement; acute renal failure requiring hemodialysis; and ICU and hospital length of stay and mortality.

### **Primary data analysis**

The Strengthening the Reporting of Observational Studies in Epidemiology (STROBE) guidelines were followed in the design and reporting of this observational study [22]. PCH and AM analyzed the data, which were summarized as number (in percentage) and median (with inter-quartile range). Missing data were coded explicitly as described and handled by using logic expression. Continuous variables were dichotomized at the median. The odds ratios and 95 % confidence intervals were computed from performing logistic regression. Statistical significance was set at 0.05 for regression analysis. Frequency of ALI was calculated per number of ED patients presenting with predisposing condition at the time of hospital admission.

In order to evaluate our secondary outcome measures, we compared hospital and ICU mortality and length of stay between ED patients at risk who developed ALI and those who did not. To determine the mortality burden attributed to the development of ALI, we performed a logistic regression analysis adjusted for the baseline Acute Physiology and Chronic Health Evaluation (APACHE II) score [23]. In addition, we described the utilization and duration of invasive and non-invasive mechanical ventilation, and performed an exploratory analysis comparing patients who did and did not develop ALI with respect to their initial vent settings, specifically tidal volume per predicted body weight, plateau pressure, and positive end expiratory pressure (PEEP). Lastly, we illustrated the frequency of ALI development for each predisposing condition, at hospital day onset, and at each hospital setting. All statistical analysis was performed in SAS 9.2 (SAS Institute, Cary, NC).

### **Sensitivity analysis**

Sites with retrospective data and extreme frequencies of ALI development were excluded, and a sensitivity analysis of the prospective cohort from the entire ED cohort was performed.

## Results and discussion

### **Results**

#### **Characteristic of study subjects**

**Figure 2:**
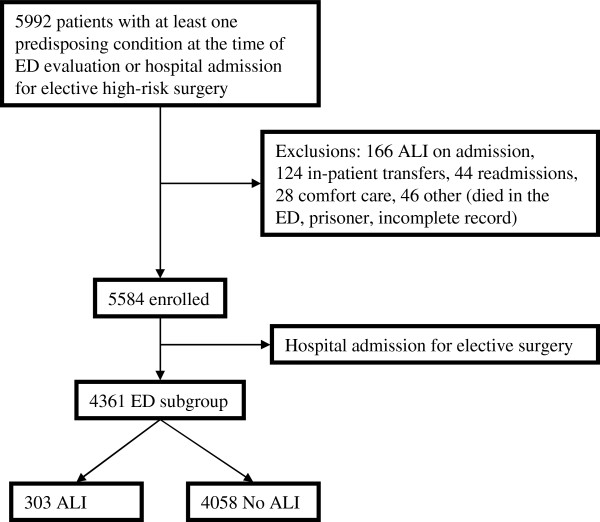
Patient enrollment flow diagram.

Twenty-two centers screened 5,992, excluded 166 patients with ALI at admission and other criteria, and enrolled 5,584 patients with at least one ALI predisposition, of which 4,361 patients were admitted from the ED (Figure 2).

### **ALI Developed within 48 H of Admission in At-Risk Patients and Markedly Increased Mortality**

**Figure 3:**
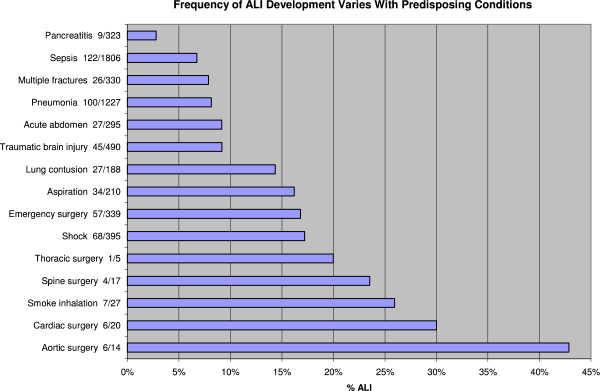
Frequency of ALI Development Varies with Predisposing Condition.

ALI developed in 303 (7.0 %) admitted ED patients with a median of 2 days, inter-quartile range (IQR) 2–5 days. Among patients who developed ALI, a subset of 198 (65.3 %) met the ARDS criteria. The follow-up to hospital discharge was complete in all patients. Baseline characteristics, severity of illness, and predisposing conditions and ALI risk modifiers differed between patients who did and those who did not develop ALI (Table 1). Patients who developed ALI were more likely male and heavier, and had a higher APACHE II score. The majority of patients had all measurements available at the time of hospital admission except for serum albumin (*n* = 2,423) and arterial pH (*n* = 1,499). As these tests are usually ordered based on clinical suspicion, missing data were considered normal (i.e., if serum albumin or arterial pH was not measured, hypoalbuminemia and acidosis were coded as absent), similarly to APACHE score calculation. The odds ratios for ALI development were highest in risk modifiers of tachypnea and a fraction of inspired oxygen 35 % or greater; and in predisposing conditions of aortic vascular, cardiac, and spinal surgeries, near-drowning, and smoke inhalation. However, the numbers of subjects in some of these categories were small compared to others. The frequency of ALI varied according to predisposing condition with the highest rate occurring after emergent aortic surgery (42.9 %) and the lowest rate occurring in pancreatitis (2.8 %) (Figure 3).

**Table 1:** Demographics, predisposing conditions, and risk modifiers in total, no ALI, ALI

**Variable**	**Total (*n* = 4361)**	**No ALI (*n* = 4058)**	**ALI (*n* = 303)**	**OR (95 % CI)**	**P-value**
**Demographics**					
Median age (Q1, Q3)	56.0 (41.0, 71.0)	56.0 (41.0, 71.0)	54.0 (41.0, 67.0)	0.99 (0.99, 1.00)	0.050
Male, no. (%)	2,422 (55.5 %)	2222 (54.8 %)	200 (66.0 %)	1.60 (1.26, 2.05)	< 0.001
Caucasian (n = 4220), no. (%),	2608 (61.8 %)	2424 (61.8 %)	184 (62.0 %)	1.01 (0.79, 1.28)	0.956
Weight (n = 3905), median (Q1, Q3)	76.5 (63.5, 91.0)	76.0 (63.5, 91.0)	83.2 (69.2, 96.0)	1.84 (1.43, 2.37)	< 0.001
PBW(n = 3551), median (Q1, Q3)	63.8 (64.7, 73.0)	63.8 (54.6, 73.0)	66.1 (56.4, 75.0)	1.42 (1.10, 1.84)	0.007
Admission source (n = 4311), no. (%)					
Home	3,331 (77.3 %)	3,125 (78.0 %)	206 (68.2 %)	1.00	
Nursing facility	338 (7.8 %)	322 (8.0 %)	16 (5.3 %)	0.75 (0.45, 1.27)	0.002
Outside ED	440 (10.2 %)	396 (9.9 %)	44 (14.6 %)	1.69 (1.20, 2.37)	0.253
Other	202 (4.7 %)	166 (4.1 %)	36 (11.9 %)	3.29 (2.23, 4.84)	< 0.001
APACHE II (Q1, Q3)	10.0 (6.0, 15.0)	9.0 (5.0, 14.0)	15.0 (10.0, 21.0)	1.11 (1.09, 1.13)	< 0.001
**Predisposing conditions**					
Shock	395 (9.1 %)	327 (8.1 %)	68 (22.4 %)	3.30 (2.46, 4.42)	< 0.001
Aspiration	210 (4.8 %)	176 (4.3 %)	34 (1.2 %)	2.79 (1.89, 4.11)	< 0.001
Sepsis	1,806 (41.4 %)	1,684 (41.5 %)	122 (40.3 %)	0.95 (0.75, 1.21)	0.674
Pancreatitis	323 (7.4 %)	314 (7.7 %)	9 (3.0 %)	0.37 (0.19, 0.72)	0.003
Pneumonia	1,227 (28.1 %)	1,127 (27.8 %)	100 (33.0 %)	1.28 (0.99, 1.64)	0.051
High-risk trauma					
Traumatic brain injury	490 (11.2 %)	445 (11.0 %)	45 (14.9 %)	1.42 (1.02, 1.97)	0.040
Smoke inhalation	27 (0.6 %)	20 (0.5 %)	7 (2.3 %)	4.78 (2.00, 11.38)	< 0.001
Near drowning	3 (0.1 %)	2 (0.1 %)	1 (0.3 %)	6.72 (0.61, 74.27)	0.120
Lung contusion	188 (4.3 %)	161 (4.0 %)	27 (8.9 %)	2.37 (1.55, 3.63)	< 0.001
Multiple fractures	330 (7.6 %)	304 (7.5 %)	26 (8.6 %)	1.16 (0.76, 1.76)	0.489
High-risk surgery					
Thoracic (noncardiac)	5 (0.1 %)	4 (0.1 %)	1 (0.3 %)	3.36 (0.37, 30.12)	0.280
Orthopedic spine	17 (0.4 %)	13 (0.3 %)	4 (1.3 %)	4.16 (1.35, 12.85)	0.013
Acute abdomen	295 (6.8 %)	268 (6.6 %)	27 (8.9 %)	1.38 (0.91, 2.09)	0.125
Cardiac surgery	20 (0.5 %)	14 (0.3 %)	6 (2.0 %)	5.84 (2.23, 15.30)	< 0.001
Aortic vascular	14 (0.3 %)	8 (0.2 %)	6 (2.0 %)	10.23 (3.53, 29.68)	< 0.001
Emergency surgery	339 (7.7 %)	282 (7.0 %)	57 (18.8 %)	3.10 (2.27, 4.24)	< 0.001
**Risk modifiers**					
Alcohol abuse	421 (9.7 %)	381 (9.4 %)	40 (13.2 %)	1.47 (1.04, 2.08)	0.031
Obesity (*n* = 3,508)	1,020 (29.1 %)	929 (28.6 %)	91 (35.0 %)	1.34 (1.03, 1.75)	0.029
Chemotherapy	158 (3.6 %)	145 (3.6 %)	13 (4.3 %)	1.21 (0.68, 2.16)	0.520
Diabetes mellitus	1,042 (23.9 %)	987 (24.3 %)	55 (18.2 %)	0.69 (0.51, 0.93)	0.016
Smoking (*n* = 4,019)					
None	2,060 (51.3 %)	1,931 (51.6 %)	129 (47.1 %)	1.00	--
Former	888 (22.1 %)	829 (22.1 %)	59 (21.5 %)	1.07 (0.78, 1.47)	0.644
Active	1,071 (26.7 %)	985 (26.3 %)	86 (31.4 %)	1.31 (0.98, 1.74)	0.089
RR (*n* = 4,137), median (Q1,Q3)	20.0 (18.0, 24.0)	20.0 (18.0, 24.0)	22.0 (18.0, 27.0)		
Tachypnea (*n* = 4,137), no. (%)	315 (7.6 %)	269 (6.8 %)	52 (18.8 %)	3.18 (2.29, 4.40)	< 0.001
SpO_2_ (*n* = 4,361), median (Q1, Q3)	97.0 (94.0, 99.0)	97.0 (94.0, 99.0)	95.0 (92.0, 98.0)		
SpO_2_ < 95 % (*n* = 4,361)	1,203 (27.6 %)	1,076 (26.5 %)	127 (41.9 %)	2.17 (1.56, 3.02)	< 0.001
FiO_2_ (*n* = 4,361), median (Q1, Q3)	0.2 (0.2, 0.3)	0.2 (0.2, 0.3)	0.4 (0.2, 1.0)		
FiO_2_ >0.35 (*n* = 4,796), no. (%)	841 (19.3 %)	688 (17.0 %)	153 (50.5 %)	4.92 (3.87, 6.25)	< 0.001
Albumin (*n* = 2,423), median (Q1, Q3)	3.5 (2.9, 4.0)	3.5 (3.0, 4.0)	3.2 (2.4, 3.7)		
Hypoalbuminemia (*n* = 2,423), no. (%)	945 (47.1 %)	838 (45.6 %)	107 (64.5 %)	2.17 (1.56, 3.02)	< 0.001
pH (*n* = 1,499), median (Q1, Q3)	7.4 (7.3, 7.4)	7.4 (7.3, 7.4)	7.3 (7.2, 7.4)		
Acidosis (pH <7.35), no. (%)	476 (45.9 %)	364 (43.3 %)	112 (56.6 %)	1.70 (1.25, 2.33)	< 0.001

PRW: Predicted body weight; RR: respiratory rate; tachypnea = RR > 30; SpO_2_: oxygen saturation; FiO_2_: fraction of inspired oxygen.

Outcome data for the study cohort are shown in Table 2. More than half of the entire cohort and 91 % of patients who developed ALI were treated in the ICU; and 31 % of the entire cohort and 95 % of patients with ALI were either invasively or non-invasively mechanically ventilated. Compared to at-risk patients who did not develop ALI, those who developed ALI were more likely to be ventilated invasively (88 % vs 19 %) and non-invasively (30 % vs 10 %), respectively. Similarly, comparing to patients who did not develop ALI, those who did had increased resource utilization as reflected in more vasopressor usage (38 % vs 8 %), a higher percentage of acute hemodialysis requirement (11 % vs 3 %), and longer ICU (9 vs 2 days) and hospital (16 vs 5 days) lengths of stays. More importantly, patients who developed ALI had increased ICU (24 % vs 3 %) and hospital (28 % vs 5 %) mortality. When adjusted for severity of illness using the APACHE II score, the development of ALI markedly increased the risk of in-hospital death by more than four-fold [OR 4.45, 95%CI (3.23, 6.14)].

**Table 2:** Hospital Course and Outcomes for Total, No ALI, and ALI

**Variable**	**Total (*n* = 4,361)**	***No ALI (n* = 4,058)**	**ALI (*n* = 303)**	**OR (95 % CI)**	**P-value**
ICU admission, no. (%)	2,320 (53.2 %)	2,043 (50.3 %)	277 (91.4 %)	10.50 (6.99, 15.77)	< 0.001
ICU LOS (*n* = 2,320), median (Q1, Q3)	2.0 (1.0, 5.0)	2.0 (0.0, 4.0)	9.0 (5.0, 17.0)	19.12 (9.79, 37.38)	< 0.001
Hospital LOS (n = 4361), median (Q1, Q3)	6.0 (3.0, 10.0)	5.0 (3.0, 9.0)	16.0 (9.0, 26.0)	8.60 (5.94, 12.36)	< 0.001
Vasopressors use, no. (%)	448 (10.3 %)	334 (8.2 %)	114 (37.6 %)	6.73 (5.20, 8.71)	< 0.001
Acute hemodialysis (*n* = 4,290), no. (%)	148 (3.5 %)	115 (2.9 %)	33 (11.0 %)	4.15 (2.76, 6.23)	< 0.001
ICU mortality, no. (%)	194 (4.5 %)	120 (3.0 %)	74 (24.4 %)	10.61 (7.71, 14.59)	< 0.001
Hospital mortality, no. (%)	272 (6.2 %)	188 (4.6 %)	84 (27.7 %)	7.90 (5.90, 10.56)	< 0.001
Mechanical ventilation (*n* = 4,223)	1,299 (30.8 %)	1,013 (25.8 %)	286 (94.7 %)	51.29 (30.85, 85.28)	< 0.001
Non-invasive (*n* = 4,146), no. (%)	470 (11.3 %)	387 (10.0 %)	83 (30.1 %)	3.87 (2.93, 5.11)	< 0.001
Non-invasive duration (*n* = 461), median (Q1, Q3)	2.0 (1.0, 5.0)	2.0 (1.0, 5.0)	3.0 (2.0, 5.5)	1.29 (0.73, 2.25)	0.379
Invasive (*n* = 4,228), no. (%)	997 (23.6 %)	730 (18.6 %)	267 (88.4 %)	33.40 (23.27, 47.94)	< 0.001
Invasive duration (*n* = 932), median (Q1, Q3)	3.0 (1.0, 8.0)	2.0 (1.0, 5.0)	8.0 (4.0, 15.0)	6.53 (4.52, 9.42)	< 0.001
TV/PBW (*n* = 768), median (Q1, Q3)	8.3 (7.4, 9.5)	8.4 (7.5, 9.7)	8.1 (7.3, 9.2)	0.70 (0.51, 0.96)	0.029
Plateau pressure (*n* = 435), median (Q1, Q3)	19.0 (16.0, 24.0)	19.0 (15.0, 23.0)	21.0 (17.0, 27.2)	1.77 (1.17, 2.69)	0.007
PEEP (*n* = 916), median (Q1, Q3)	5.0 (5.0, 5.5)	5.0 (5.0, 5.0)	5.0 (5.0, 8.0)	2.30 (1.32, 4.01)	0.003
Mode: Volume control	762 (83.9 %)	561 (86.4 %)	201 (77.6 %)	1.00	--
Mode: Pressure control	111 (12.2 %)	63 (9.7 %)	48 (18.5 %)	2.13 (1.41, 3.20)	0.010

ICU: Intensive care unit; LOS: length of stay; TV: tidal volume; PBW: predicted body weight; PEEP: peak end-expiratory pressure.

**Figure 4:**
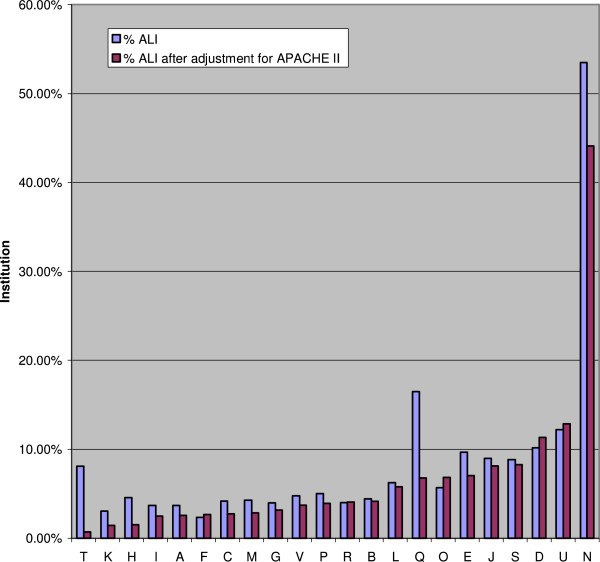
Frequency of ALI Development Varies by Institution.

The variation in the frequency of ALI development among the 22 study sites is illustrated in Figure 4. Even after adjustment for each site’s APACHE II score, a significant variation in the frequency of ALI development remained. By excluding the one outlier (44 %), the frequency of ALI development varied from 0.7 to 12.8 %.

**Figure 5:**
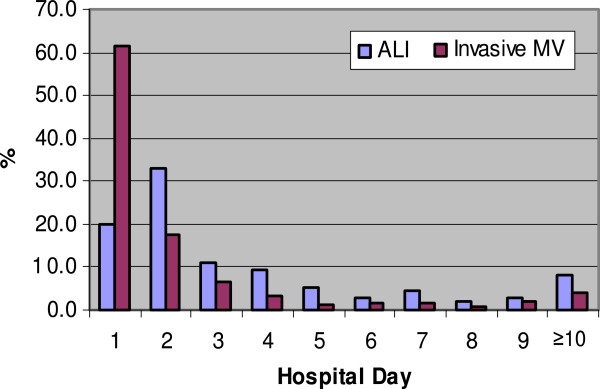
Percentage of ALI Development and Invasive Mechanical Ventilation Onset by Hospital Day.

**Figure 6:**
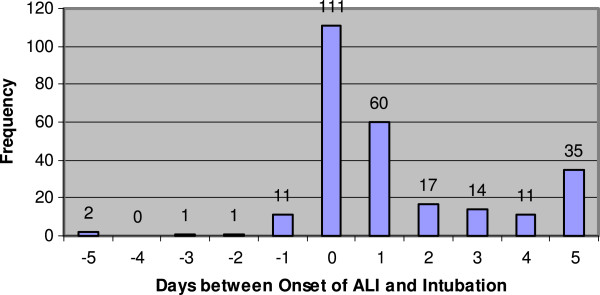
Days between Onset of ALI Development and Invasive Mechanical Ventilation.

Since the data set is fairly large, the utilization of invasive mechanical ventilation and onset of ALI development were explored. Figure 5 provides a description of ALI development and initiation of invasive mechanical ventilation by hospital day. The majority of patients who developed ALI and who received invasive mechanical ventilation did so within the first 2 days after hospital admission. As illustrated in Figure 6, the majority of patients who developed ALI had initiation of invasive mechanical ventilation on the day of ALI onset. Unfortunately, no data were recorded regarding the exact timing (in hours and minutes) and the reason for initiation of invasive mechanical ventilation with respect to the onset of ALI development. Although relatively few patients developed ALI prior to initiation of invasive mechanical ventilation, many developed ALI a day or more after initiation of invasive mechanical ventilation.

### **Sensitivity analyses**

When data from the two non-US study sites were removed (*n* = 4,233) from the analysis (see Additional file 1: Appendix 3), ALI developed in 266 (6.3 %) patients with a median of 2 days (IQR 2–5 days) after admission to the hospital. The frequency of ALI varied according to predisposing condition with the highest rate of ALI occurring after aortic surgery (36.4 %) and the lowest rate occurring in pancreatitis (2.9 %). Baseline characteristics, severity of illness, predisposing conditions and ALI risk modifiers, and secondary outcomes remained different between patients who did and those who did not develop ALI. However, there were no significant differences in the group characteristics from the US study sites when comparing to the entire cohort.

In addition, when data from the three retrospective US study sites were removed (*n* = 3,981) from our descriptive analysis (see Additional file 1: Appendix 4), ALI developed in 241 (6.1 %) patients with a median of 2 days (IQR 2–4 days) after admission to the hospital. The frequency of ALI varied according to predisposing condition with the highest rate of ALI occurring after aortic surgery (40 %) and the lowest rate occurring in pancreatitis (2.4 %). Baseline characteristics, severity of illness, predisposing conditions and ALI risk modifiers, and secondary outcomes maintained their differences between patients who did and did not develop ALI. However, there were no significant differences in the group characteristics when comparing the prospective US sites and the entire US cohort.

### **Effects of initial ventilator settings on the development of ALI**

Since there is growing evidence to suggest that a lung protective strategy with low tidal volume ventilation may be protective in at-risk patients, we explored the effect of the initial vent setting on ALI development [24,25]. We evaluated the initial tidal volume divided by predicted body weight (TV/PBW in cc/kg) in 768 patients who underwent invasive mechanical ventilation (Table 2). Comparing those who did not, patients who developed ALI received a significant lower initial TV/PBW [8.1 vs 8.4, OR 0.70 (95 % CI 0.51-0.96), *p* = 0.029] but had a significantly higher plateau pressure [21 vs 19, OR 1.77 (95 % CI 1.17-2.69), *p* = 0.007] and peak end-expiratory pressure (PEEP) [OR 2.30 (95 % CI 1.32-4.01), *p* = 0.003]. Regarding the mode of mechanical ventilation, a significantly higher proportion of patients who developed ALI was initiated on pressure control ventilation [OR 2.13 (95 % CI 1.41-3.2), *p* = 0.01] and non-invasive ventilation [OR 3.87 (95 % CI 2.93-5.11), *p* < 0.001] compared to those who did not develop ALI; in contrast, no significant difference was found in those who underwent initial volume control ventilation.

### **Limitations**

Our study carries the limitations of observational cohort studies and those inherent to clinical research with ALI: inter-observer reliability of portable chest X-ray interpretation, ALI imitators, and consistency in determining exclusion of left atrial hypertension as the principal cause of pulmonary edema [26-28]. We instituted a mandatory structured training in ALI assessment and held the site-principal investigators responsible for quality control. These measures were intended to mitigate these limitations.

Regarding the variation in the frequency in ALI development among the study sites, two non-US sites had the highest rate and could be explained by differences in their health-care delivery system, specifically emergency medical care and critical care services, and possibly population and environmental factors. When the data from these two non-US centers were excluded, no significant difference in the frequency of ALI development (*p* = 0.216) was found between the US sites. In addition, the vast majority of patients were enrolled prospectively ensuring close follow-up and reducing the risk of misclassification from medical record review. When data from those centers enrolling retrospectively were excluded, no significant difference in the frequency of ALI development (*p* = 0.665) was found comparing the prospective US sites to the entire US cohort. Hence, although a significant variation in the frequency of ALI development across institutions was found, the US sites and the prospectively enrolling US sites were not significantly different when each subgroup was compared to the entire cohort.

### **Discussion**

Even though ALI is classified as a rare disease [29], it is a major public health concern. It is, however, unclear how much if variations in initial management of ALI-prone patients by emergency physicians contribute to its development. To our knowledge, this is the largest detailed study of a cohort of hospitalized adult ED patients at risk for ALI development. The strengths of this study include the large sample size from a geographically diverse population of patients at both academic and community hospitals. Using routinely available clinical data, we identified ED patients at risk for ALI development early in the course of their illness. The early identification of predisposing conditions and risk modifiers as well as subsequent interventions in the ED and ICU may potentially prevent disease development by minimizing or avoiding secondary insults.

Interestingly, in our study we found that ALI developed with lower frequencies than previously reported following conditions recognized to predispose patients at risk for ALI such as: aspiration, pneumonia, sepsis, and trauma [30,31]. This may be explained by the fact that our patients were enrolled early (in the ED) without any signs of ALI (with at least one predisposing factor present) and we excluded those who developed ALI within 6 h of ED presentation, while those other studies enrolled patients upon ICU admission. Consistent with our findings, a recent study by Ferguson et al. showed that 7 % of patients with sepsis, 2 % of patients with pancreatitis, 10 % of patients with pneumonia, and 15 % of patients with witnessed aspiration developed ALI [32]. Similarly, the majority of patients with predisposing conditions never developed ALI, and many were not admitted to the ICU.

Our inclusion criteria required the presence of at least one ALI risk factor at the time of hospital admission, potentially missing the patients who acquire a predisposing condition and received a secondary injury later in the hospital stay. We cannot rule out that a minority of our patients identified as high risk were already progressing to develop full-blown ALI at the time of enrollment, although the exclusion of those who developed ALI within 6 h of ED presentation intended to minimize this possibility. However, we do believe that earlier identification of such patients would also be of benefit, and could help limit the progression of ALI development and improve patient outcomes by alerting providers to make efforts to limit second-hit exposures. To support this notion, a population-based study in Olmsted County, Minnesota, has recently shown a steady decline in ARDS incidence. This was attributable entirely to a reduced incidence of hospital-acquired ARDS and suggests that recent improvements in prevention, early recognition, and critical care delivery may in part be responsible for this [33].

From Olmsted County’s experience, major system changes within the hospital throughout the years were progressively made. These include electronic medical records with computerized order entry to monitor data and institute decision support, restrictive transfusion protocol with leukoreduction and male donor predominant plasma transfusion, respiratory therapy protocol on limiting initial volume according to predicted body weight on all patients, increased staffing of intensivists with 24-h on site, sepsis resuscitation protocols and teams, rapid response teams, standardization of inpatient pneumonia care, and staff education and training. Regarding potential interventions that may affect the development of ALI, a checklist for lung injury prevention (CLIP) has been proposed and developed by experts in the field [34]. The CLIP domains (and elements) consist of routine ICU practices of morbidity prevention and include: respiratory support (lung protective strategies, minimizing oxygen toxicity) [12,24,25]; aspiration precautions (rapid sequence intubation, head-of-bed elevation, oral care with chlorhexidine) [35-37]; infection control (early and appropriate antibiotic therapy, source control, prevention of nosocomial infection transmission) [38]; fluid management (early fluid resuscitation in severe sepsis and septic shock, fluid restriction after shock resolution) [39,40]; transfusion management (restrictive red blood cell transfusion threshold, transfusion guidelines for blood products) [40,41]; and communication (validated structured handoffs such as SBAR: situation, background, assessment, and recommendation) [42]. As a continuum in the care of the critically ill starting in the ED and transitioning to the ICU, all of these domains and many of these proposed elements are being followed to a certain extent, but inconsistently as reflected in our own experiential observations and practice variations. However, such a proposed checklist will require validation for its utility in adherence to best practices and ALI prevention.

## Conclusions

Many ED patients who are hospitalized have risk factors for ALI development. In this cohort, we found 7 % of ED patients with at least one predisposing condition developed ALI, and there is variation in the frequency of ALI development across study sites. In addition, more resources are utilized in patients who do develop ALI, and more importantly, ALI significantly increased the patient’s risk of death in the hospital. Hence, is there a role for emergency physicians in the management of patients at risk for ALI development?

Currently, pre-planned ancillary studies are ongoing to explore potential differences in development of ALI at different hospital settings, in different disease-related groups, and their specific treatment modalities. In addition, acute lung injury prevention trials are being proposed. Further research is also warranted to develop a prediction model to identify hospitalized ED patients at risk of ALI development at an early stage in their illness.

## Abbreviations

AECC: American-European consensus conference; ALI: acute lung injury; APACHE: acute physiology and chronic health evaluation; ARDS: acute respiratory distress syndrome; CLIP: checklist for lung injury prevention; ED: emergency department; ICU: intensive care units; LIPS: lung injury prediction score; PEEP: positive end expiratory pressure; PBW: predicted body weight; TV: tidal volume; USCIITG-LIPS 1: United States Critical Injury and Illness Trials Group-Lung Injury Prevention Study 1; VALI: ventilator-associated lung injury.

## Competing interests

The STAR Center provided internal funding (Dr. Frendl), research staff, and biostatistical support, Brigham and Women’s Hospital, Boston, MA. Dr. Gajic is supported in part by grants from the National Heart, Lung, and Blood Institute HL78743-01A1; National Center for Research Resources 1 KL2 RR024151. Dr. Gentile is supported in part by grants from the National Institute of Neurological Disorders and Stroke 5U10NS059039-04. The rest of the authors have no disclosures or conflict of interest.

## Authors' contributions

PCH, MCE, OG, and NTG conceived the study, designed the review, and supervised the conduct of the review and data collection. GF obtained research funding. PCH, AM, and OG extracted and managed the data and performed quality control of the data. AM provided statistical advice on study design, and all authors analyzed the data. PCH drafted the manuscript, and all authors provided significant contributions to its revision. PCH takes responsibility for the paper as whole. All authors read and approved the final manuscript.

## Supplementary Material

Additional file 1 **Web files Appendices**[43-94]. Click here for file
