# Supplementary material for: Towards prevention of acute lung injury: frequency and outcomes of emergency department patients at-risk – a multicenter cohort study
Source: Int J Emerg Med. 2012 May 27;5:22. doi: 10.1186/1865-1380-5-22 (PMC3598496; doi:10.1186/1865-1380-5-22)
Supplement: Additional file 1 — Web files Appendices[43-94]. [file 1865-1380-5-22-S1.pdf]

Web files Appendices

1. **Material and Methods: Study Flow**
2. **Material and Methods: Definition of clinical variables (including references)**
3. **Table 1 & 2 comparing US versus Non-US study sites**
4. **Table 1 & 2 comparing Prospective versus Retrospective study sites in the US**
5. **Acknowledgement**

<u>**Appendix 1**</u>

Materials and Methods:

Study Flow

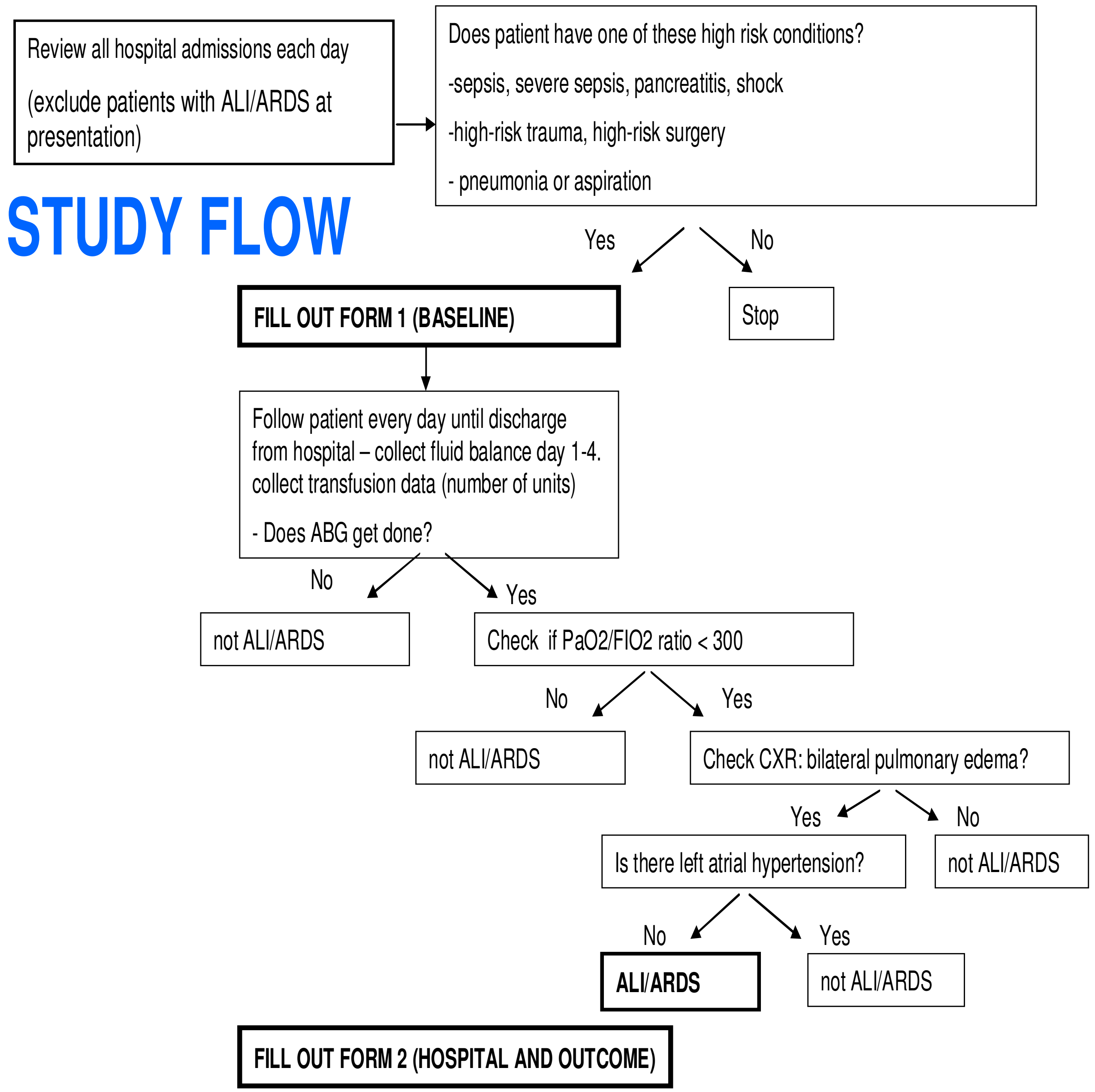

Appendix 2

Materials and Methods:

Definitions of clinical variables

Alcohol abuse: Known diagnosis of chronic alcoholism or a previous admission for alcohol detoxification or alcohol withdrawal; daily alcohol consumption of >14 drinks a week; or >5 drinks binges[1].

Tobacco exposure: Patients or surrogates were asked about used of tobacco products in the number of cigarettes and also the amount per day and the years of smoking, in order to report pack-years. United States Department of Health and Human Services, Substance Abuse and Mental Health Services Administration (http://www.samhsa.gov/index.aspx). Former smoker is defined if the patient quits 30 or more days before admission

Diabetes mellitus: Documentation of diabetes in the clinical record based on the 2007 American Diabetes Association Statement of the Expert Committee on the Diagnosis and Classification of Diabetes Mellitus[7]:

1. Symptoms of diabetes plus casual plasma glucose concentration >200 mg/dl. Casual is defined as any time of day without regard to time since last meal. The classic symptoms of diabetes include polyuria, polydipsia, and unexplained weight loss.

OR

2. FPG >126 mg/dl. Fasting is defined as no caloric intake for at least 8 h.

OR

3. 2-h postload glucose > 200 mg/dl during an OGTT. The test should be performed as described by WHO, using a glucose load containing the equivalent of 75 g anhydrous glucose dissolved in water.

Chronic interstitial lung disease: As defined by the American Thoracic Society/European Respiratory Society International Multidisciplinary Consensus Classification of the Idiopathic Interstitial Pneumonias[10] as a group of disorders of known causes (collagen vascular disease, environmental or drug related) as well as disorders of unknown cause. The latter include idiopathic interstitial pneumonias (IIPs), granulomatous lung disorders (e.g., sarcoidosis), and other forms of interstitial lung disease (ILD) including lymphangioleiomyomatosis (LAM), pulmonary Langerhans' cell histiocytosis/histiocytosis X (HX), and eosinophilic pneumonia. The most important distinction among the idiopathic interstitial pneumonias is that between idiopathic pulmonary fibrosis and the other interstitial pneumonias (IPs), which include nonspecific interstitial pneumonia (a provisional term), desquamative interstitial pneumonia, pneumonia, and lymphocytic interstitial pneumonia. respiratory bronchiolitis-associated interstitial lung disease, acute interstitial pneumonia, cryptogenic organizing pneumonia.

Cirrhosis: Cirrhosis represents a late stage of progressive hepatic fibrosis characterized by distortion of the hepatic architecture and the formation of regenerative nodules. The gold standard for diagnosis of cirrhosis is with a liver biopsy, however, liver biopsy is not necessary if the clinical, laboratory, and radiologic data strongly suggest the presence of cirrhosis. An example would be a patient with ascites, severe coagulopathy, and a shrunken nodular appearing liver on ultrasonography[12].

Cancer, lymphoma and leukemias: as defined by the National Cancer Institute in the Terms & Definitions MP/H Coding Rules (http://www.cancer.gov/search/results.aspx)

• **Carcinoma** - cancer that begins in the skin or in tissues that line or cover internal organs.

• **Sarcoma** - cancer that begins in bone, cartilage, fat, muscle, blood vessels, or other connective or supportive tissue.

• **Leukemia** - cancer that starts in blood-forming tissue such as the bone marrow and causes large numbers of abnormal blood cells to be produced and enter the blood.

• **Lymphoma and myeloma** - cancers that begin in the cells of the immune system.

• **Central nervous system cancers** - cancers that begin in the tissues of the brain and spinal cord.

• **Metastasis**: The spread of cancer from one part of the body to another. A tumor formed by cells that have spread is called a "metastatic tumor" or a "metastasis." The metastatic tumor contains cells that are like those in the original (primary) tumor.

AIDS: Defined by the CDC[20] as all patients in categories A3, B3, C1-C3 from the table below are reported as AIDS based upon prior AIDS-indicator conditions and/or a CD4 cell count <200/mm3. AIDS-indicator conditions include three new entries added to the 1987 case definition: recurrent bacterial pneumonia, invasive cervical cancer, and pulmonary tuberculosis. Symptomatic conditions not included in category C that (a) are attributed to HIV infection or indicate a defect in cell-mediated immunity or (b) are conditions considered to have a clinical course or to require management that is complicated by HIV infection. Examples of B conditions include but are not limited to baciliary angiomatosis; thrush; vulvovaginal candidiasis that is persistent, frequent or poorly responsive to therapy; cervical dysplasia (moderate or severe); cervical carcinoma in situ; constitutional symptoms such as fever (38.5°C) or diarrhea for more than one month; oral hairy leukoplakia; and herpes zoster involving two episodes or more than one dermatome.

Immunosuppression: defined as therapy with immunosuppressants, chemotherapy, radiation or long term/recent high dose of steroids; or active leukemia, lymphoma or AIDS[22].

Heart Failure: Documentation of heart failure in the clinical record as defined by the ACC/AHA 2005 Guideline Update for the Diagnosis and Management of Chronic Heart Failure in the Adult: a report of the American College of Cardiology/American Heart Association Task Force on Practice Guidelines(Writing Committee to Update the 2001 Guidelines for the Evaluation and Management of Heart Failure): developed in collaboration with the American College of Chest Physicians and the International Society for Heart and Lung Transplantation: endorsed by the Heart Rhythm Society[6] Heart failure is a complex clinical syndrome that can result from any structural or functional cardiac disorder that impairs the ability of the ventricle to fill with or eject blood. HF is defined as a clinical syndrome that is characterized by specific symptoms (dyspnea and fatigue) in the medical history and signs (edema, rales) on the physical examination. There is no single diagnostic test for HF because it is largely a clinical diagnosis that is based on a careful history and physical examination. The clinical syndrome of HF may result from disorders of the pericardium, myocardium, endocardium, or great vessels, but majority of patients with HF has symptoms due to an impairment of LV myocardial function. Heart failure may be associated with a wide spectrum of LV functional abnormalities, which may range from patients with normal LV size and preserved EF to those with severe dilatation and/or markedly reduced EF. In most patients, abnormalities of systolic and diastolic dysfunction coexist, regardless of EF.

COPD: Documentation in the clinical record of COPD based on the Global Strategy for the Diagnosis, Management, and Prevention of Chronic Obstructive Pulmonary Disease NHLBI/WHO Global Initiative for Chronic Obstructive Lung Disease (GOLD) Workshop Summary[8]: COPD is a disease state characterized by airflow limitation that is not fully

reversible. The airflow limitation is usually both progressive and associated with an abnormal inflammatory response of the lungs to noxious particles or gases. A diagnosis of COPD should be considered in any patient who has symptoms of cough, sputum production, or dyspnea, and/or a history of exposure to risk factors for the disease. The diagnosis is confirmed by spirometry. The presence of a post-bronchodilator $FEV_1 < 80\%$ of the predicted value in combination with an $FEV_1/FVC < 70\%$ confirms the presence of airflow limitation that is not fully reversible. Where spirometry is unavailable, the diagnosis of COPD should be made using all available tools. Clinical symptoms and signs, such as abnormal shortness of breath and increased forced expiratory time, can be used to help with the diagnosis. A low peak flow is consistent with COPD, but has poor specificity because it can be caused by other lung diseases and by poor performance. In the interest of improving the diagnosis of COPD, every effort should be made to provide access to standardized spirometry. Chronic cough and sputum production often precede the development of airflow limitation by many years, although not all individuals with cough and sputum production go on to develop COPD.

Chronic kidney disease: The National Kidney Foundation - Kidney Disease Outcomes Quality Initiative (NKF-K/DOQI) workgroup has defined CKD as the following [13], which has been accepted internationally [14]: the presence of markers of kidney damage for ≥3 months, as defined by structural or functional abnormalities of the kidney with or without decreased glomerular filtration rate (GFR), that can lead to decreased GFR, manifest by either pathological abnormalities or other markers of kidney damage, including abnormalities in the composition of blood or urine, or abnormalities in imaging tests; or the presence of GFR <60 mL/min/1.73 m2 for ≥3 months, with or without other signs of kidney damage as described above.

Pneumonia: be defined according to three different categories as established by the 2005 International Sepsis Forum Consensus Conference on Definitions of Infection in the Intensive

Care Unit: *Microbiologically confirmed*: The patient must have a new or progressive radiographic infiltrate, along with a high clinical suspicion of pneumonia plus a definite cause established by the recovery of a probable etiologic agent from a) an uncontaminated specimen (blood, pleural fluid, transtracheal aspirate, or transthoracic aspirate); b) the recovery from respiratory secretions of a likely pathogen that does not colonize the upper airways (e.g., *Mycobacterium tuberculosis*, *Legionella* species, influenza virus, or *Pneumocystis jiroveci (carinii*); c) recovery of a likely/possible respiratory pathogen in high concentrations using quantitative cultures of a lower respiratory tract sample (endotracheal aspirate, BAL, or protected specimen brush); or d) positive serology. *Probable*: The patient must have a new or progressive radiographic infiltrate along with a high clinical suspicion of pneumonia plus detection (by staining or culture) of a likely pulmonary pathogen in respiratory secretions (expectorated sputum, endotracheal or bronchoscopic aspirate, or quantitatively cultured bronchoscopic BAL fluid or brush catheter specimen), but in concentrations below the diagnostic threshold, or the presence of a negative lower respiratory tract culture if collected within 72 hrs after starting a new antibiotic regimen. *Possible*: Abnormal chest radiograph of uncertain cause, in a patient with a low or moderate clinical suspicion of pneumonia, but with microbiological or serological evidence of definite or probable pneumonia[23].

Given the timing of our evaluation (hospital admission) the microbiologic proof not be possible and not be required and a practical CDC definition be applied:

1) Chest radiographs showing new or progressive infiltrate, consolidation, cavitation or pleural effusion

AND

2) Either of:

   i. New onset purulent sputum and/or change in its character,

OR

ii. IF AVAILABLE:

1. positive blood culture or isolation of pathogen from specimen obtained by transtracheal space, bronchial brushing or biopsy and/or isolation of virus or viral antigen detection from respiratory secretions
2. diagnostic single antibody titer (IgM) or fourfold increase in sera (IgG) for pathogen
3. histopathological evidence of pneumonia

Sepsis: Suspected or documented infection in the presence of more than one of the following clinical manifestations: **(1)** a body temperature greater than 38°C or less than 36°C; **(2)** a heart rate greater than 90 beats per minute; **(3)** tachypnea, manifested by a respiratory rate greater than 20 breaths per minute, or hyperventilation, as indicated by a PaCO2 of less than 32 mm Hg; and **(4)** an alteration in the white blood cell count, such as a count greater than 12,000/cu mm, a count less than 4,000/cu mm, or the presence of more than 10 percent immature neutrophils ("bands")[24].

Shock: We defined shock as recommended by the 2006 International Consensus Conference on Hemodynamic Monitoring in Shock and Implications for Management[25]: Presence of hypotension with evidence of inadequate tissue perfusion on physical examination (altered mental status not explained by other causes other than the hemodynamic status and urine output less than 0.5 ml/Kg/min). Hypotension was defined as a systolic blood pressure (SBP) $<$ 90mmHg, or SBP decrease of 40 mmHg from baseline, or mean arterial pressure (MAP) $<$ 65mmHg[25]. We expanded the definition of shock in the absence of hypotension when shock is suggested by history and physical examination; according to the consensus recommendation[25]. In this case, markers of inadequate perfusion were used and defined as follows: central venous oxygen saturation (ScvO2) or mixed venous oxygen saturation (SvO2) less than 70%[26], blood

lactate levels greater than 4 mmol/L[26] in the absence of known acute or chronic liver disease, increased base deficit < -4[26]and blood pH less than 7.32[26].

Septic shock: Sepsis in the presence of shock[24] that is defined as hypotension with evidence of inadequate tissue perfusion: systolic blood pressure (SBP) < 90mmHg, or SBP decrease of 40 mmHg from baseline, or mean arterial pressure (MAP) < 65mmHg[25].

Hypovolemic shock: presence of shock as previously described, in the setting of clinical documented volume loss and resolution of hypotension and normalization of markers of tissue hypoperfusion after resuscitation with fluid or blood products or surgical intervention to obtain vascular control when appropriate[25, 27]. There must not be convincing evidence for a primary alternative diagnosis of cardiogenic or distributive shock[27].

Cardiogenic shock: presence of shock as previously described, in the setting of clinical documented inadequate tissue perfusion due to cardiac dysfunction. The most common etiology is an acute myocardial infarction (MI) with left ventricular failure, but it can also be caused by mechanical complications, such as acute mitral regurgitation or ventricular septal defect, hypertrophic cardiomyopathy, valvular disease, or myocarditis[28, 29].

Aspiration: witnessed or suggestive history of inhalation of food or regurgitated gastric contents[30].

Acute pancreatitis: Defined by the Practice Guidelines in Acute Pancreatitis as two of the following three features: 1) abdominal pain characteristic of acute pancreatitis, 2) serum amylase and/or lipase >/ 3 times the upper limit of normal, and 3) characteristic findings of acute pancreatitis on CT scan[31].

Trauma: presence of lung contusion, blast injury, fracture of two or more long bones, traumatic brain injury and smoke inhalation[35].- Near drowning: Submersion accident with at least temporary survival (near drowning), or water rescue or removal of victim from water (save)[32].

- Heat stroke: is defined as a core body temperature in excess of 40.5ºC (105ºF) with associated central nervous system dysfunction in the setting of a large environmental heat load that cannot be dissipated[33].

High risk surgery: defined as any of the following procedures[36, 37]:

- All cardiac and aortic vascular procedures (excluding endovascular procedures)
- Noncardiac thoracic surgery including esophageal and pulmonary (excluding thoracoscopic surgery)
- Acute abdomen
- Orthopedic spine surgeries

Mechanical ventilation(MV): Invasive and non-invasive mechanical ventilation for more than 1 hour, except for post operative patients requiring MV for less than 12 hours after the surgery and patients who use chronic non-invasive ventilation treatment for obstructive sleep apnea (OSA) were excluded [46, 47].

Glasgow Coma Scale: defined according to the original description by Teasdale and collaborators[49].

Inspired oxygen concentration FIO_2 and arterial oxygen tension PaO_2: When arterial blood gases are not available, FIO_2 and PaO_2 was estimated from oxygen saturation (SaO_2) and FIO_2 according to Rice at al[52] .An SaO_2/ FIO_2value of 235 corresponds with P/F ratio of 200 while S/F

value of 315 corresponds with P/F ratio of 300. In non-intubated patients approximate FIO_2 were estimated based on oxygen flow and device type.

References:

1. Moss M, Bucher B, Moore FA, Moore EE, Parsons PE. The role of chronic alcohol abuse in the development of acute respiratory distress syndrome in adults. In: Journal of the American Medical Association; 1996:50-4.
2. Selzer ML. The Michigan alcoholism screening test: the quest for a new diagnostic instrument. Am J Psychiatry 1971;127(12):1653-8.
3. Charlson M, Szatrowski TP, Peterson J, Gold J. Validation of a combined comorbidity index. J Clin Epidemiol 1994;47(11):1245-51.
4. Chobanian AV, Bakris GL, Black HR, et al. Seventh report of the Joint National Committee on Prevention, Detection, Evaluation, and Treatment of High Blood Pressure. Hypertension 2003;42(6):1206-52.
5. Chobanian AV, Bakris GL, Black HR, et al. The Seventh Report of the Joint National Committee on Prevention, Detection, Evaluation, and Treatment of High Blood Pressure: the JNC 7 report. JAMA 2003;289(19):2560-72.
6. Hunt SA. ACC/AHA 2005 guideline update for the diagnosis and management of chronic heart failure in the adult: a report of the American College of Cardiology/American Heart Association Task Force on Practice Guidelines (Writing Committee to Update the 2001 Guidelines for the Evaluation and Management of Heart Failure). J Am Coll Cardiol 2005;46(6):e1-82.
7. Diagnosis and classification of diabetes mellitus. Diabetes Care 2007;30 Suppl 1:S42-7.
8. Pauwels RA, Buist AS, Ma P, Jenkins CR, Hurd SS. Global strategy for the diagnosis, management, and prevention of chronic obstructive pulmonary disease: National Heart, Lung, and Blood Institute and World Health Organization Global Initiative for Chronic Obstructive Lung Disease (GOLD): executive summary. Respir Care 2001;46(8):798-825.

9. Expert Panel Report 3 (EPR-3): Guidelines for the Diagnosis and Management of Asthma-Summary Report 2007. J Allergy Clin Immunol 2007;120(5 Suppl):S94-138.

10. American Thoracic Society/European Respiratory Society International Multidisciplinary Consensus Classification of the Idiopathic Interstitial Pneumonias. This joint statement of the American Thoracic Society (ATS), and the European Respiratory Society (ERS) was adopted by the ATS board of directors, June 2001 and by the ERS Executive Committee, June 2001. Am J Respir Crit Care Med 2002;165(2):277-304.

11. Sonnenberg A, Everhart JE. Health impact of peptic ulcer in the United States. Am J Gastroenterol 1997;92(4):614-20.

12. Anthony PP, Ishak KG, Nayak NC, Poulsen HE, Scheuer PJ, Sobin LH. The morphology of cirrhosis. Recommendations on definition, nomenclature, and classification by a working group sponsored by the World Health Organization. J Clin Pathol 1978;31(5):395-414.

13. K/DOQI clinical practice guidelines for chronic kidney disease: evaluation, classification, and stratification. Am J Kidney Dis 2002;39(2 Suppl 1):S1-266.

14. Levey AS, Eckardt KU, Tsukamoto Y, et al. Definition and classification of chronic kidney disease: a position statement from Kidney Disease: Improving Global Outcomes (KDIGO). Kidney Int 2005;67(6):2089-100.

15. Alpert JS, Thygesen K, Antman E, Bassand JP. Myocardial infarction redefined--a consensus document of The Joint European Society of Cardiology/American College of Cardiology Committee for the redefinition of myocardial infarction. J Am Coll Cardiol 2000;36(3):959-69.

16. Thygesen K, Alpert JS, White HD. Universal definition of myocardial infarction. J Am Coll Cardiol 2007;50(22):2173-95.

17. Hirsch AT, Haskal ZJ, Hertzer NR, et al. ACC/AHA 2005 guidelines for the management of patients with peripheral arterial disease (lower extremity, renal, mesenteric, and abdominal aortic): executive summary a collaborative report from the American Association for Vascular Surgery/Society for Vascular Surgery, Society for Cardiovascular Angiography and Interventions, Society for Vascular Medicine and Biology, Society of Interventional Radiology, and the ACC/AHA Task Force on Practice Guidelines (Writing Committee to Develop Guidelines for the Management of Patients With Peripheral Arterial Disease) endorsed by the American Association of Cardiovascular and Pulmonary Rehabilitation; National Heart, Lung, and Blood Institute; Society for Vascular Nursing; TransAtlantic Inter-Society Consensus; and Vascular Disease Foundation. J Am Coll Cardiol 2006;47(6):1239-312.

18. Adams HP, Jr., del Zoppo G, Alberts MJ, et al. Guidelines for the early management of adults with ischemic stroke: a guideline from the American Heart Association/American Stroke Association Stroke Council, Clinical Cardiology Council, Cardiovascular Radiology and Intervention Council, and the Atherosclerotic Peripheral Vascular Disease and Quality of Care Outcomes in Research Interdisciplinary Working Groups: The American Academy of Neurology affirms the value of this guideline as an educational tool for neurologists. Circulation 2007;115(20):e478-534.

19. Knopman DS, DeKosky ST, Cummings JL, et al. Practice parameter: diagnosis of dementia (an evidence-based review). Report of the Quality Standards Subcommittee of the American Academy of Neurology. Neurology 2001;56(9):1143-53.

20. Karon JM, Buehler JW, Byers RH, et al. Projections of the number of persons diagnosed with AIDS and the number of immunosuppressed HIV-infected persons--United States, 1992-1994. MMWR Recomm Rep 1992;41(RR-18):1-29.

21. Steinman TI, Becker BN, Frost AE, et al. Guidelines for the referral and management of patients eligible for solid organ transplantation. Transplantation 2001;71(9):1189-204.

22. Knaus WA, Wagner DP, Draper EA, et al. The APACHE III prognostic system. Risk prediction of hospital mortality for critically ill hospitalized adults. Chest 1991;100(6):1619-36.

23. Calandra T, Cohen J. The international sepsis forum consensus conference on definitions of infection in the intensive care unit. Crit Care Med 2005;33(7):1538-48.

24. American College of Chest Physicians/Society of Critical Care Medicine Consensus Conference: definitions for sepsis and organ failure and guidelines for the use of innovative therapies in sepsis. Crit Care Med 1992;20(6):864-74.

25. Antonelli M, Levy M, Andrews PJ, et al. Hemodynamic monitoring in shock and implications for management. International Consensus Conference, Paris, France, 27-28 April 2006. Intensive Care Med 2007;33(4):575-90.

26. Rivers E, Nguyen B, Havstad S, et al. Early goal-directed therapy in the treatment of severe sepsis and septic shock. N Engl J Med 2001;345(19):1368-77.

27. Gutierrez G, Reines HD, Wulf-Gutierrez ME. Clinical review: hemorrhagic shock. Crit Care 2004;8(5):373-81.

28. Hollenberg SM, Kavinsky CJ, Parrillo JE. Cardiogenic shock. Ann Intern Med 1999;131(1):47-59.

29. Hochman JS, Boland J, Sleeper LA, et al. Current spectrum of cardiogenic shock and effect of early revascularization on mortality. Results of an International Registry. SHOCK Registry Investigators. Circulation 1995;91(3):873-81.

30. Marik PE. Aspiration pneumonitis and aspiration pneumonia. N Engl J Med 2001;344(9):665-71.

31. Banks PA. Practice guidelines in acute pancreatitis. Am J Gastroenterol 1997;92(3):377-86.

32. Sachdeva RC. Near drowning. Crit Care Clin 1999;15(2):281-96.

33. Bouchama A, Knochel JP. Heat stroke. N Engl J Med 2002;346(25):1978-88.

34. Voelkel NF. High-altitude pulmonary edema. N Engl J Med 2002;346(21):1606-7.

35. Derdak S. Acute respiratory distress syndrome in trauma patients. J Trauma 2007;62(6 Suppl):S58.

36. Arozullah AM, Khuri SF, Henderson WG, Daley J. Development and validation of a multifactorial risk index for predicting postoperative pneumonia after major noncardiac surgery. Ann Intern Med 2001;135(10):847-57.

37. Arozullah AM, Daley J, Henderson WG, Khuri SF. Multifactorial risk index for predicting postoperative respiratory failure in men after major noncardiac surgery. The National Veterans Administration Surgical Quality Improvement Program. Ann Surg 2000;232(2):242-53.

38. Zimmerman JE, Kramer AA, McNair DS, Malila FM. Acute Physiology and Chronic Health Evaluation (APACHE) IV: hospital mortality assessment for today's critically ill patients. Crit Care Med 2006;34(5):1297-310.

39. Rubenfeld GD, Angus DC, Pinsky MR, Curtis JR, Connors AF, Jr., Bernard GR. Outcomes research in critical care: results of the American Thoracic Society Critical Care Assembly Workshop on Outcomes Research. The Members of the Outcomes Research Workshop. Am J Respir Crit Care Med 1999;160(1):358-67.

40. Kleinman S, Caulfield T, Chan P, et al. Toward an understanding of transfusion-related acute lung injury: statement of a consensus panel. Transfusion 2004;44(12):1774-89.

41. Toy P, Popovsky MA, Abraham E, et al. Transfusion-related acute lung injury: definition and review. Crit Care Med 2005;33(4):721-6.

42. McGregor JC, Rich SE, Harris AD, et al. A systematic review of the methods used to assess the association between appropriate antibiotic therapy and mortality in bacteremic patients. Clin Infect Dis 2007;45(3):329-37.

43. Kumar A, Roberts D, Wood KE, et al. Duration of hypotension before initiation of effective antimicrobial therapy is the critical determinant of survival in human septic shock. Crit Care Med 2006;34(6):1589-96.

44. Dellinger RP, Carlet JM, Masur H, et al. Surviving Sepsis Campaign guidelines for management of severe sepsis and septic shock. Crit Care Med 2004;32(3):858-73.

45. Jimenez MF, Marshall JC. Source control in the management of sepsis. Intensive Care Med 2001;27 Suppl 1:S49-62.

46. Esteban A, Anzueto A, Frutos F, et al. Characteristics and outcomes in adult patients receiving mechanical ventilation: a 28-day international study. JAMA 2002;287(3):345-55.

47. Vincent JL, Akca S, De Mendonca A, et al. The epidemiology of acute respiratory failure in critically ill patients(*). Chest 2002;121(5):1602-9.

48. Vincent JL, Moreno R, Takala J, et al. The SOFA (Sepsis-related Organ Failure Assessment) score to describe organ dysfunction/failure. On behalf of the Working Group on Sepsis-Related Problems of the European Society of Intensive Care Medicine. Intensive Care Med 1996;22(7):707-10.

49. Teasdale G, Jennett B. Assessment of coma and impaired consciousness. A practical scale. Lancet 1974;2(7872):81-4.

50. Sessler CN, Gosnell MS, Grap MJ, et al. The Richmond Agitation-Sedation Scale: Validity and reliability in adult intensive care unit patients. American Journal of Respiratory and Critical Care Medicine 2002;166(10):1338-44.

51. Ely EW, Inouye SK, Bernard GR, et al. Delirium in mechanically ventilated patients: Validity and reliability of the Confusion Assessment Method for the intensive care unit (CAM-ICU). Journal of the American Medical Association 2001;286(21):2703-10.

52. Rice TW, Wheeler AP, Bernard GR, Hayden DL, Schoenfeld DA, Ware LB. Comparison of the SpO2/FiO2 Ratio and the PaO2/FiO2 Ratio in Patients with Acute Lung Injury or Acute Respiratory Distress Syndrome. Chest 2007.

Appendix 3

Table 1: Demographics, Predisposing conditions, and Risk modifiers				
**Variable**	**Total (n=4361)**	**US sites (n=4233)**	**Non US sites (n=128)**	**P-value**
**Demographics**				
Median age (Q1, Q3)	56.0 (41.0, 71.0)	56.0 (41.0, 71.0)	57.0 (38.5, 68.5)	0.396
Male, no. (%)	2422 (55.5%)	2332 (55.1%)	90 (70.3%)	< 0.001
Caucasian (n=4220), no. (%),	2608 (61.8%)	2608 (63.7%)	0 (0.0%)	< 0.001
Weight (n=3905), median (Q1, Q3)	76.5 (63.5, 91.0)	76.5 (63.5, 91.6)	76.5 (65.0, 88.0)	0.523
PBW(n=3551), median (Q1, Q3)	63.8 (64.7, 73.0)	63.8 (54.6, 73.0)	65.9 (56.9, 70.5)	0.874
Admission source (n=4311), no. (%)				< 0.001
Home	3331 (77.3%)	3249 (77.7%)	82 (64.1%)	
Nursing facility	338 (7.8%)	338 (8.1%)	0 (0.0%)	
Outside ED	440 (10.2%)	423 (10.1%)	17 (13.%)	
Other	202 (4.7%)	173 (4.1%)	29 (22.7%)	
APACHE II (Q1, Q3)	10.0 (6.0, 15.0)	10.0 (5.0, 15.0)	13.5 (7.0, 19.0)	< 0.001
**Predisposing conditions**				
Shock	395 (9.1%)	391 (9.2%)	4 (3.1%)	0.018
Aspiration	210 (4.8%)	202 (4.8%)	8 (6.3%)	0.442
Sepsis	1806 (41.4%)	1795 (42.4%)	11 (8.6%)	< 0.001
Pancreatitis	323 (7.4%)	308 (7.3%)	15 (11.7%)	0.059
Pneumonia	1227 (28.1%)	1189 (28.1%)	38 (29.7%)	0.692
High risk trauma				
Traumatic brain injury	490 (11.2%)	474 (11.2%)	16 (12.5%)	0.646
Smoke inhalation	27 (0.6%)	27 (0.6%)	0 (0.0%)	1.000
Near drowning	3 (0.1%)			
Lung contusion	188 (4.3%)	179 (4.2%)	9 (7.0%)	0.124
Multiple fractures	330 (7.6%)	307 (7.3%)	23 (18.0%)	< 0.001
High risk surgery				
Thoracic (noncardiac)	5 (0.1%)	5 (0.1%)	0 (0.0%)	1.000
Orthopedic spine	17 (0.4%)	14 (0.3%)	2 (2.34%)	0.012
Acute abdomen	295 (6.8%)	273 (6.5%)	22 (17.2%)	< 0.001
Cardiac surgery	20 (0.5%)	12 (0.3%)	8 (6.3%)	< 0.001
Aortic vascular	14 (0.3%)	11 (0.3%)	3 (2.3%)	< 0.001
Emergency surgery	339 (7.7%)	302 (7.1%)	37 (28.9%)	< 0.001
**Risk Modifiers**				
Alcohol abuse	421 (9.7%)	417 (9.9%)	4 (3.1%)	0.011
Obesity (n=3508)	1020 (29.1%)	996 (29.5%)	24 (18.8%)	0.009
Chemotherapy	158 (3.6%)	157 (3.7%)	1 (0.8%)	0.091

Diabetes mellitus	1042 (23.9%)	1020 (24.1%)	22 (17.2%)	0.071
Smoking (n=4019)				0.222
None	2060 (51.3%)	2005 (51.5%)	55 (43.7%)	
Former	888 (22.1%)	856 (22.0%)	32 (25.4%)	
Active	1071 (26.7%)	1032 (26.5%)	39 (31.0%)	
RR (n=4137), median (Q1,Q3)	20.0 (18.0, 24.0)	20.0 (18.0, 24.0)	20.0 (16.0, 24.0)	0.057
Tachypnea (n=4137), no. (%)	315 (7.6%)	304 (7.6%)	11 (8.7%)	0.632
SpO2 (n=4361), median (Q1, Q3)	97.0 (94.0, 99.0)	97.0 (94.0, 99.0)	97.0 (93.0, 98.0)	0.541
SpO2 >95% (n=4361)	1203 (27.6%)	1166 (27.6%)	37 (28.9%)	0.734
FiO2 (n=4361), median (Q1, Q3)	0.2 (0.2, 0.3)	0.2 (0.2, 0.3)	0.4 (0.2, 0.5)	< 0.001
FiO2 >0.35 (n=4796), no. (%)	841 (19.3%)	757 (17.9%)	76 (59.4%)	< 0.001
Albumin (n=2423), median (Q1, Q3)	3.5 (2.9, 4.0)	3.5 (2.9, 4.0)	3.8 (3.3, 4.1)	0.173
Hypoalbuminemia (n=2423), no. (%)	945 (47.1%)	939 (47.3%)	6 (30.0%)	0.123
pH (n=1499), median (Q1, Q3)	7.4 (7.3, 7.4)	7.4 (7.3, 7.4)	7.4 (7.3, 7.4)	0.043
Acidosis (pH <7.35), no. (%)	476 (45.9%)	435 (46.9%)	41 (37.3%)	0.056

PRW: Predicted Body Weight; RR: Respiratory Rate; Tachypnea = RR > 30; SpO2: Oxygen Saturation; FiO2: Fraction of Inspired Oxygen

Table 2: Hospital Course and Outcomes

Variable	Total (n=4361)	US sites (n=4233)	Non US sites (n=128)	P-value
ALI	303 (7.0%)	266 (6.3%)	37 (28.9%)	< 0.001
ICU admission, no. (%)	2320 (53.2%)	2217 (52.4%)	103 (80.5%)	< 0.001
ICU LOS (n=2320), median (Q1, Q3)	2.0 (1.0, 5.0)	2.0 (1.0, 5.0)	5.0 (2.0, 17.0)	< 0.001
Hospital LOS (n=4361), median (Q1, Q3)	6.0 (3.0, 10.0)	6.0 (3.0, 10.0)	13.5 (7.0, 26.5)	< 0.001
Vasopressors use, no. (%)	448 (10.3%)	408 (9.6%)	40 (31.3%)	< 0.001
Acute hemodialysis (n=4290), no. (%)	148 (3.5%)	140 (3.4%)	8 (6.3%)	0.084
ICU mortality, no. (%)	194 (4.5%)	174 (4.1%)	20 (15.6%)	< 0.001
Hospital mortality, no. (%)	272 (6.2%)	249 (5.9%)	23 (18.0%)	< 0.001
Mechanical ventilation (n=4223)	1299 (30.8%)	1206 (29.5%)	93 (72.7%)	< 0.001
Invasive (n=4228), no. (%)	997 (23.6%)	906 (22.1%)	91 (71.1%)	< 0.001
Invasive duration (n=932), median (Q1, Q3)	3.0 (1.0, 8.0)	3.0 (1.0, 8.0)	4.0 (1.0, 19.0)	0.011
Mode: Volume Control	762 (83.9%)	691 (84.5%)	71 (78.9%)	0.099
Mode: Pressure Control	111 (12.2%)	94 (11.5%)	17 (18.9%)	
TV/PBW (n=768), median (Q1, Q3)	8.3 (7.4, 9.5)	8.2 (7.3, 9.5)	8.6 (7.9, 9.6)	0.031
TV/PBW (n=768) > 8, no. (%)	456 (59.4%)	391 (57.7%)	65 (72.2%)	0.016
TV/PBW (n=768) 6-8, no. (%)	272 (35.4%)	248 (36.6%)	24 (26.7%)	
TV/PBW (n=768) < 6, no. (%)	40 (5.2%)	39 (5.8%)	1 (1.1%)	
Plateau Pressure (n=435), median (Q1, Q3)	19.0 (16.0, 24.0)	20.0 (17.0, 25.0)	15.9 (14.0, 18.0)	< 0.001
PEEP, (n=916), median (Q1, Q3)	5.0 (5.0, 5.5)	5.0 (5.0, 5.0)	5.0 (5.0, 6.0)	0.018
Non-invasive (n=4146), no. (%)	470 (11.3%)	460 (11.5%)	10 (7.9%)	0.211
Non-invasive duration (n=461), median (Q1, Q3)	2.0 (1.0, 5.0)	2.0 (1.0, 5.0)	2.0 (2.0, 3.0)	0.745

ICU: Intensive Care Unit; LOS: Length of Stay; TV: Tidal Volume; PBW: Predicted Body Weight; PEEP: Peak End-Expiratory Pressure

Appendix 4

Table 1: Demographics, Predisposing conditions, and Risk modifiers				
**Variable**	**US sites (n=4233)**	**Prospective (n=3981)**	**Retrospective (n=252)**	**P-value**
**Demographics**				
Median age (Q1, Q3)	56.0 (41.0, 71.0)	56.0 (42.0, 72.0)	52.0 (40.5, 65.0)	0.011
Male, no. (%)	2332 (55.1%)	2163 (54.3%)	169 (67.1%)	< 0.001
Caucasian (n=4220), no. (%),	2608 (63.7%)	2429 (63.1%)	179 (73.7%)	< 0.001
Weight (n=3905), median (Q1, Q3)	76.5 (63.5, 91.6)	76.4 (63.5, 91.6)	76.8 (64.0, 91.2)	0.958
PBW(n=3551), median (Q1, Q3)	63.8 (54.6, 73.0)	63.8 (54.6, 73.0)	66.0 (56.9, 73.7)	0.018
Admission source (n=4311), no. (%)				< 0.001
Home	3249 (77.7%)	3079 (78.2%)	170 (69.7%)	
Nursing facility	338 (8.1%)	325 (8.3%)	13 (5.3%)	
Outside ED	423 (10.1%)	383 (9.7%)	40 (16.4%)	
Other	173 (4.1%)	152 (3.9%)	21 (8.6%)	
APACHE II (Q1, Q3)	10.0 (5.0, 15.0)	10.0 (6.0, 14.0)	11.0 (5.0, 17.0)	0.295
**Predisposing conditions**				
Shock	391 (9.2%)	353 (8.9%)	38 (15.1%)	0.001
Aspiration	202 (4.8%)	130 (3.3%)	72 (28.6%)	< 0.001
Sepsis	1795 (42.4%)	1717 (43.1%)	78 (31.0%)	< 0.001
Pancreatitis	308 (7.3%)	294 (7.4%)	14 (5.6%)	0.278
Pneumonia	1189 (28.1%)	1105 (27.8%)	84 (33.3%)	0.056
High risk trauma				
Traumatic brain injury	474 (11.2%)	472 (11.9%)	2 (0.8%)	< 0.001
Smoke inhalation	27 (0.6%)	21 (0.5%)	6 (2.4%)	0.004
Near drowning				
Lung contusion	179 (4.2%)	152 (3.8%)	27 (10.7%)	< 0.001
Multiple fractures	307 (7.3%)	273 (6.9%)	34 (13.5%)	< 0.001
High risk surgery				
Thoracic (noncardiac)	5 (0.1%)	5 (0.1%)	0 (0.0%)	1.000
Orthopedic spine	14 (0.3%)	14 (0.4%)	0 (0.0%)	1.000
Acute abdomen	273 (6.5%)	258 (6.5%)	15 (6.0%)	0.741
Cardiac surgery	12 (0.3%)	12 (0.3%)	0 (0.0%)	1.000
Aortic vascular	11 (0.3%)	10 (0.3%)	1 (0.4%)	0.491
Emergency surgery	302 (7.1%)	285 (7.2%)	17 (6.8%)	0.805
**Risk Modifiers**				
Alcohol abuse	417 (9.9%)	360 (9.0%)	57 (22.6%)	< 0.001
Obesity (n=3508)	996 (29.5%)	939 (29.5%)	57 (28.4%)	0.722
Chemotherapy	157 (3.7%)	154 (3.9%)	3 (1.2%)	0.029

Diabetes mellitus	1020 (24.1%)	973 (24.4%)	47 (18.7%)	0.037
Smoking (n=4019)				< 0.001
None	2005 (51.5%)	1896 (51.9%)	109 (46.0%)	
Former	856 (22.0%)	839 (23.0%)	17 (7.2%)	
Active	1032 (26.5%)	921 (25.2%)	111 (46.8%)	
RR (n=4137), median (Q1,Q3)	20.0 (18.0, 24.0)	20.0 (18.0, 24.0)	26.0 (19.0, 32.0)	< 0.001
Tachypnea (n=4137), no. (%)	304 (7.6%)	280 (7.1%)	24 (30.0%)	< 0.001
SpO2 (n=4361), median (Q1, Q3)	97.0 (94.0, 99.0)	97.0 (94.0, 98.0)	96.0 (93.0, 98.0)	< 0.001
SpO2 >95% (n=4361)	1166 (27.6%)	1070 (26.9%)	96 (38.1%)	< 0.001
FiO2 (n=4361), median (Q1, Q3)	0.2 (0.2, 0.3)	0.2 (0.2, 0.3)	0.5 (0.3, 1.0)	< 0.001
FiO2 >0.35 (n=4796), no. (%)	757 (17.9%)	669 (16.8%)	88 (34.9%)	< 0.001
Albumin (n=2423), median (Q1, Q3)	3.5 (2.9, 4.0)	3.5 (3.0, 4.0)	2.8 (2.5, 3.5)	< 0.001
Hypoalbuminemia (n=2423), no. (%)	939 (47.3%)	881 (46.3%)	58 (71.6%)	< 0.001
pH (n=1499), median (Q1, Q3)	7.4 (7.3, 7.4)	7.4 (7.3, 7.4)	7.4 (7.3, 7.4)	0.982
Acidosis (pH <7.35), no. (%)	435 (46.9%)	383 (46.9%)	52 (46.9%)	0.995

PRW: Predicted Body Weight; RR: Respiratory Rate; Tachypnea = RR > 30; SpO2: Oxygen Saturation; FiO2: Fraction of Inspired Oxygen

Table 2: Hospital Course and Outcomes				
**Variable**	**US sites (n=4233)**	**Prospective (n=3981)**	**Retrospective (n=252)**	**P-value**
ALI	266 (6.3%)	241 (6.1%)	25 (9.9%)	0.014
ICU admission, no. (%)	2217 (52.4%)	2137 (53.7%)	80 (31.8%)	< 0.001
ICU LOS (n=2320), median (Q1, Q3)	2.0 (1.0, 5.0)	2.0 (0.0, 5.0)	4.0 (3.0, 8.0)	< 0.001
Hospital LOS (n=4361), median (Q1, Q3)	6.0 (3.0, 10.0)	5.0 (3.0, 10.0)	7.0 (4.0, 14.0)	< 0.001
Vasopressors use, no. (%)	408 (9.6%)	382 (9.6%)	26 (10.3%)	0.707
Acute hemodialysis (n=4290), no. (%)	140 (3.4%)	129 (3.3%)	11 (4.4%)	0.363
ICU mortality, no. (%)	174 (4.1%)	161 (4.0%)	13 (5.2%)	0.388
Hospital mortality, no. (%)	249 (5.9%)	233 (5.9%)	16 (6.4%)	0.745
Mechanical ventilation (n=4223)	1206 (29.5%)	1090 (27.6%)	116 (83.5%)	< 0.001
Invasive (n=4228), no. (%)	906 (22.1%)	810 (20.5%)	96 (69.1%)	< 0.001
Invasive duration (n=932), median (Q1, Q3)	3.0 (1.0, 8.0)	3.0 (1.0, 7.0)	4.0 (2.0, 9.0)	0.006
Mode: Volume Control	691 (84.5%)	596 (82.6%)	95 (99.0%)	< 0.001
Mode: Pressure Control	94 (11.5%)	93 (12.9%)	1 (1.0%)	
TV/PBW (n=768), median (Q1, Q3)	8.2 (7.3, 9.5)	8.3 (7.3, 9.6)	8.0 (7.4, 9.1)	0.230
TV/PBW (n=768) > 8, no. (%)	391 (57.7%0	348 (58.3%)	43 (53.1%)	0.556
TV/PBW (n=768) 6-8, no. (%)	248 (36.6%)	214 (35.9%)	34 (42.0%)	
TV/PBW (n=768) < 6, no. (%)	39 (5.8%)	35 (5.9%)	4 (4.9%)	
Plateau Pressure (n=435), median (Q1, Q3)	20.0 (17.0, 25.0)	21.0 (17.0, 26.0)	20.0 (17.0, 24.0)	0.661
PEEP, (n=916), median (Q1, Q3)	5.0 (5.0, 5.0)	5.0 (5.0, 5.0)	5.0 (5.0, 6.0)	0.008
Non-invasive (n=4146), no. (%)	460 (11.5%)	425 (10.8%)	35 (44.3%)	< 0.001
Non-invasive duration (n=461), median (Q1, Q3)	2.0 (1.0, 5.0)	2.0 (1.0, 4.0)	5.0 (3.0, 13.0)	< 0.001
ICU: Intensive Care Unit; LOS: Length of Stay; TV: Tidal Volume; PBW: Predicted Body Weight; PEEP: Peak End-Expiratory Pressure				

Appendix 5: Acknowledgement

USCIITG LIPS1 participating centers and corresponding investigators

Mayo Clinic Rochester, Minnesota: Adil Ahmed MD, Ognjen Gajic MD, Michael Malinchoc MS, Daryl J Kor MD, Bekele Afessa, MD, Rodrigo Cartin-Ceba, MD, Departments of Internal Medicine, Health Sciences Research, and Anesthesiology
University of Missouri, Columbia: Ousama Dabbagh, MD, MS, FCCP, FASSM, Assistant Professor of Clinical Medicine, Associate Fellowship Program Director, Diplomat, American Board of Sleep Medicine; Nena Kallenbach, Administrative Assistant
University of Michigan, Ann Arbor: Pauline K. Park, MD, FACS, FCCS, Co-Director, Surgical Intensive Care Unit, Associate Professor, Surgery, Julie Harris, Clinical Research Coordinator; Lena Napolitano, MD; Krishnan Raghavendran, MBBS; Robert C. Hyzy, MD; James Blum, MD; Christy Dean
University of Texas Southwestern Medical Center in Dallas, Texas: Adebola Adesanya, MD; Srikanth Hosur, MD; Victor Enoh, MD, Department of Anesthesiology, Division of Critical Care Medicine
University of Medicine and Dentistry of New Jersey: Steven Y. Chang, PhD, MD, Assistant Professor, MICU Director, Pulmonary and Critical Care Medicine; Amee Patrawalla, MD, MPH; Marie Elie, MD
Brigham and Women's Hospital: Peter C. Hou, MD, Jonathan M. Barry, BA, Erica Kao, Ian Shempp, BS, Atul Malhotra, MD, Jason Cohen, Gyorgy Frendl, MD, PhD, Departments of Emergency Medicine, Surgery, Internal Medicine, and Anesthesiology Perioperative and Pain Medicine, Division of Burn, Trauma, and Surgical Critical Care
Wright State University Boonshoft School of Medicine & Miama Valley Hospital: Harry Anderson, III, MD, Professor of Surgery; Kathryn Tchorz, MD, Associate Professor of Surgery; Mary C. McCarthy, MD, Professor of Surgery; David Uddin, PhD, DABCC, CIP, Director of Research
Wake Forest University Health Sciences, Winston-Salem, NC: J. Jason Hoth, MD, Assistant Professor of Surgery; Barbara Yoza, PhD, Study Coordinator
University of Pennsylvania: Mark Mikkelsen, MD, MSCE, Assistant Professor of Medicine, Pulmonary, Allergy and Critical Care Division; Jason D. Christie, MD; David F. Gaieski, MD; Paul Lanken, MD; Nuala Meyer, MD; Chirag Shah, MD
Temple University School of Medicine: Nina T. Gentile, MD, Associate Professor and Director, Clinical Research, Department of Emergency Medicine, Temple University School of Medicine; Karen Stevenson, MD, Resident, Department of Emergency Medicine; Brent Freeman, BS, Research Coordinator; Sujatha Srinivasan, MD, Resident, Department of Emergency Medicine
Mount Sinai School of Medicine: Michelle Ng Gong, MD, MS, Assistant Professor, Pulmonary, Critical Care and Sleep Medicine, Department of Medicine
Beth Israel Deaconess Medical Center, Boston, Massachusetts: Daniel Talmor, MD, Director of Anesthesia and Critical Care, Associate Professor of Anaesthesia, Harvard Medical School; S. Patrick Bender MD; Mauricio Garcia MD
Massachusetts General Hospital Harvard Medical School: Ednan Bajwa, MD, MPH, Assistant Professor; Atul Malhotra, MD, Associate Professor; B. Taylor Thompson, Associate Professor; David C. Christiani, MD, MPH, Professor
University of Washington, Harborview: Timothy R. Watkins, MD, Acting Instructor, Department of Medicine, Division of Pulmonary and Critical Care Medicine; Steven Deem, MD; Miriam Treggiari, MD, MPH
Mayo Clinic Jacksonville: Emir Festic, MD; Augustine Lee, MD; John Daniels, MD
Akdeniz University, Antalyia, Turkey: Melike Cengiz, MD, PhD; Murat Yilmaz, MD
Uludag University, Bursa, Turkey: Remzi Iscimen, MD
Bridgeport Hospital, Yale New Haven Health: David Kaufman, MD, Section Chief, Pulmonary, Critical Care & Sleep Medicine, Medical Director, Respiratory Therapy
Emory University: Annette Esper, MD; Greg Martin, MD
University of Illinois at Chicago: Ruxana Sadikot, MD, MRCP
University of Colorado: Ivor Douglas, MD

Johns Hopkins University: Jonathan Sevransky, MD, MHS, Assistant Professor of Medicine, Medical Director, JHBMC MICU

We would also like to acknowledge help and support of Rob Taylor (Vanderbilt University, Nashville, TX) and Joseph J Wick (Mayo Clinic) for the availability and maintenance of REDcap database.
